# An epidermoid cyst of the uvula causing dyspnea in an infant: A case report

**DOI:** 10.1097/MD.0000000000022573

**Published:** 2020-11-13

**Authors:** Hye Rin Lim, Hyong Ho Cho, Hyung Chae Yang, Hong Chan Kim

**Affiliations:** Department of Otolaryngology-Head and Neck Surgery, Chonnam National University Medical School and Chonnam National University Hospital, Gwangju, South Korea.

**Keywords:** epidermoid cyst, thumbprint sign, uvula cyst

## Abstract

**Rationale:**

Congenital epidermoid cysts are benign deformities that rarely affect the uvula. A uvular epidermoid cyst is painless and slow-growing. Most such cysts are asymptomatic and rarely cause oral dysfunction.

**Patient concerns:**

We present the case of a 10-month-old infant with dyspnea caused by a mass in the uvula.

**Diagnosis:**

The patient was diagnosed with a uvular epidermoid cyst via neck soft tissue X-ray and flexible laryngoscopy.

**Interventions:**

Emergency surgery was performed.

**Outcomes:**

The patient recovered immediately after the operation and was discharged 1 day later.

**Lessons:**

In an infant with a uvula cyst, early surgical treatment may be needed to prevent symptoms, such as dyspnea, requiring emergency treatment.

## 1 Introduction

Epidermoid cysts are aberrant epithelial components of ectodermal tissues; they develop during the fetal period or are acquired via trauma or surgery. Uvular cysts are rare. Faulder was the first to report a child with a uvular cyst and a few cases have since been described.^[1]^ Most cysts were asymptomatic and incidentally found. To date, no epidermoid uvular cyst causing dyspnea has been reported. We report the case of a 10-month-old infant with dyspnea caused by a mass at the uvular tip, and we review the literature.

## 2 Case report

**Figure 1:**
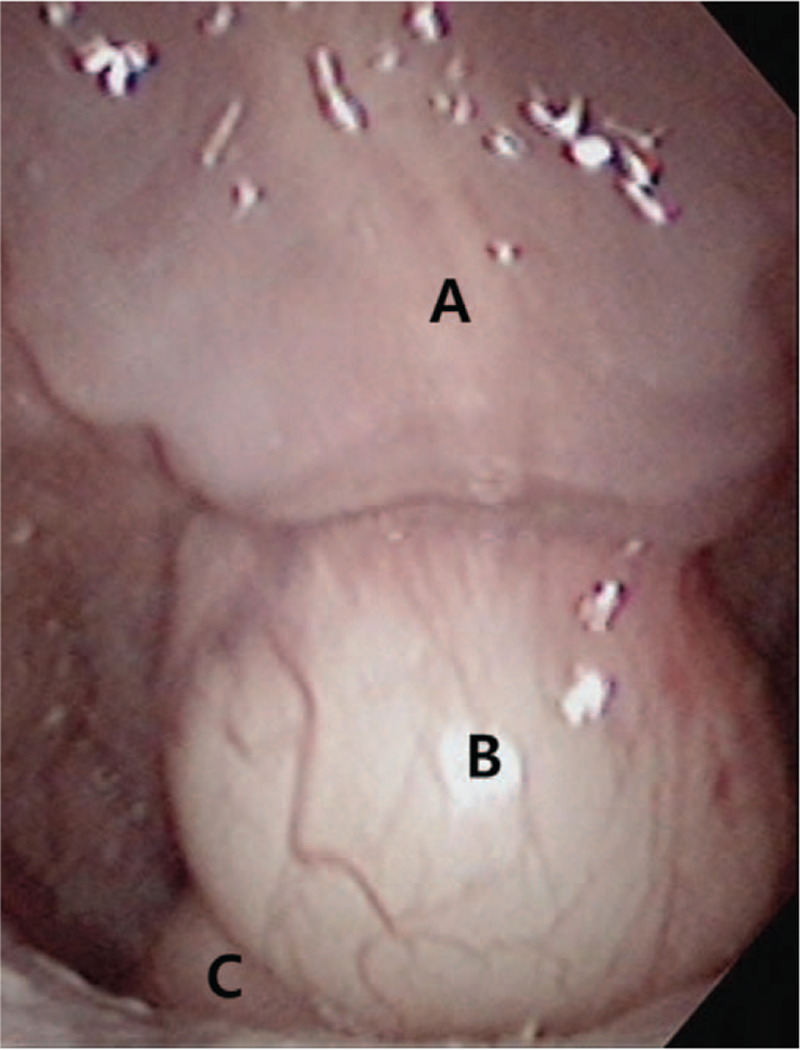
The epiglottis is compressed by the mass at the uvular tip because the uvular soft tissue became elongated during inspiration. A: uvula. B: epidermoid cyst. C: epiglottis.

**Figure 2:**
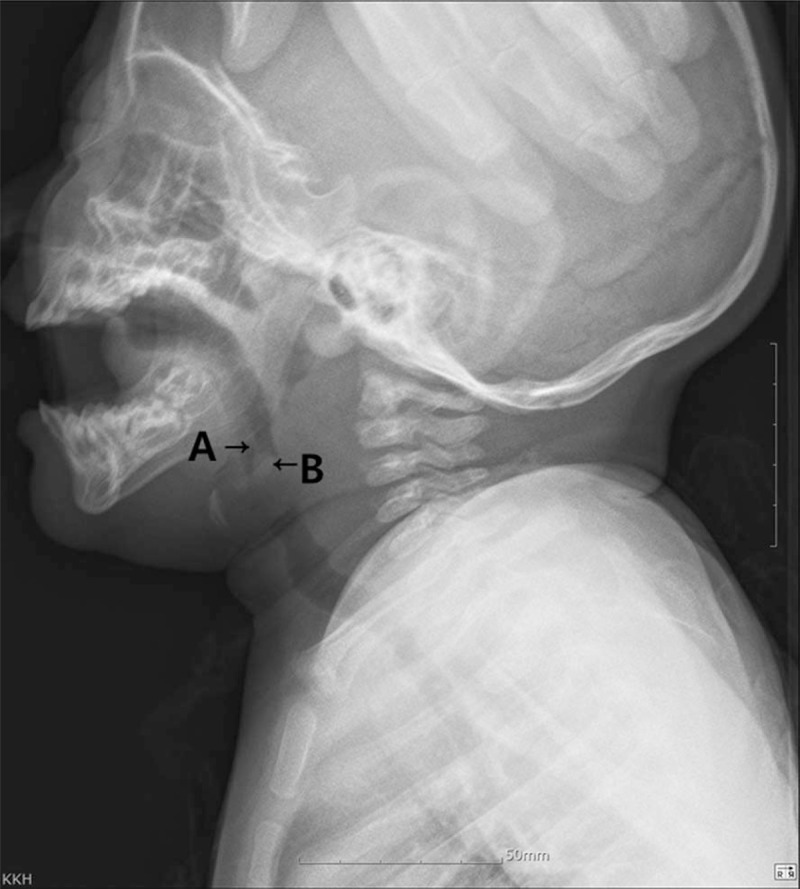
A neck soft tissue X-ray reveals the upper airway to be obstructed by the uvular cyst. A: uvula. B: epidermoid cyst.

A 10-month-old infant developed aggravated dyspnea during an upper respiratory tract infection and visited our emergency room. The patient appeared to have developed normally and had no history of feeding difficulty, dyspnea, or congenital abnormalities. Severe rhinorrhea and nasal obstruction allowed oral breathing only. Inspiration was poorer than expiration; he exhibited intermittent cyanosis. His symptoms were aggravated when lying down. Oral cavity examination using a tongue depressor revealed a well-circumscribed, round yellowish mass at the tip of an elongated uvula. On flexible laryngoscopy, the epiglottis was compressed by the mass; the uvular soft tissue became elongated during inspiration (Fig. 1). A neck soft tissue X-ray revealed a thumbprint sign and diffuse thickening of the prevertebral soft tissue (Fig. 2). Although the epiglottis was not swollen, the airway was obstructed by the cyst. We diagnosed the patient with a uvular epidermoid cyst.

**Figure 3:**
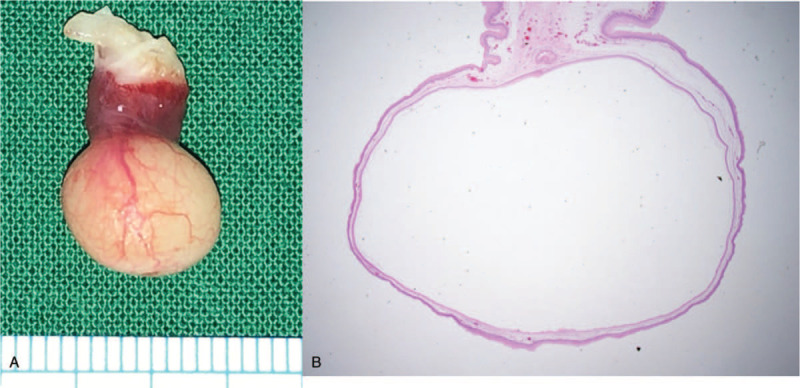
The specimen is a 2 × 1 × 0.5 cm well-circumscribed, yellowish, submucosal cystic mass. The cyst wall lining is composed of stratified squamous epithelium and the cyst contains abundant keratin flakes. The cyst wall reveals no evidence of any dermal appendage.

The cyst was removed with the child under general anesthesia induced by oral intubation; intubation was very difficult because the cyst covered the vocal cords. We pulled the uvula to expose the cyst and removed it. The specimen was a 2 ×1 × 0.5 cm, well-circumscribed, yellowish, submucosal cystic mass. The histopathological diagnosis was a benign keratinous cyst (Fig. 3). The patient recovered immediately after the operation and was discharged 1 day later. The patient is under follow-up and the uvula is healing; no recurrence has been noted 6 months postoperatively.

## 3 Discussion

Epidermoid uvular cysts are benign developmental malformations arising from abnormal epithelial constituents of ectodermal tissue formed during the fetal period.^[2]^ Cysts may be acquired or congenital.^[3]^ Factors causing acquired cysts include trauma, inflammation, and surgical complications. However, in infants, cysts are usually congenital.^[2,3]^ From weeks 6–8 of gestation, the palatal shelves migrate toward each other and eventually unite.^[4]^ Failure of this process during soft palate and uvular development can leave remnants in the midline; these may become cystic.^[4,5]^ During the fetal period, such aberrant ectodermal entrapment in the uvular area reactivates the cleaved cells, which then develop into epidermoid cysts.

Cysts can arise anywhere in the body; the incidence in the head-and-neck region is 7.0%. Within this region, the most common locations are the orbit (46.6%), followed by the floor of the mouth and submental region (23.3%), the nose (12.6%), the neck (10.7%), and the lips (2.9%).^[2]^ Only a few cases of epidermoid uvular cysts have been reported; such cysts are extremely rare. Uvular cysts are usually asymptomatic, and most are discovered incidentally. Some neonates and infants may present with impaired suckling and swallowing.^[6]^

Total surgical excision of oral cavity cysts is required. Fine-needle aspiration and fenestration reduce cystic contents but may cause infection, pain, and exacerbation.^[7]^ Our patient presented with dyspnea, and the X-ray features were similar to those of acute supraglottitis. No prior report of a uvular cyst causing dyspnea in an infant has been reported. In this patient, the upper respiratory infection rendered nasal breathing impossible. It triggered uvula swelling and increased the size of the cyst until the airway was blocked.

In conclusion, although there are no symptoms in an infant with a uvula cyst, cysts of this type can swell at any time after infection and cause dyspnea. Therefore, early surgical treatment may be required to prevent the need for emergency intervention.

## Acknowledgments

We would like to thank Editage (www.editage.com) for English language editing.

## Author contributions

**Supervision:** Hong Chan Kim, Hyong Ho Cho.

**Visualization:** Hyung Chae Yang.

**Writing – original draft:** Hye Rin Lim.

**Writing – review & editing:** Hong Chan Kim.
